# Heterosexual Transmission of Subtype C HIV-1 Selects Consensus-Like Variants without Increased Replicative Capacity or Interferon-α Resistance

**DOI:** 10.1371/journal.ppat.1005154

**Published:** 2015-09-17

**Authors:** Martin J. Deymier, Zachary Ende, Angharad E. Fenton-May, Dario A. Dilernia, William Kilembe, Susan A. Allen, Persephone Borrow, Eric Hunter

**Affiliations:** 1 Emory Vaccine Center at Yerkes National Primate Research Center, Emory University, Atlanta, Georgia, United States of America; 2 Nuffield Department of Medicine, University of Oxford, Oxford, United Kingdom; 3 Zambia-Emory HIV Research Project, Lusaka, Zambia; 4 Department of Pathology and Laboratory Medicine, Emory University, Atlanta, Georgia, United States of America; King's College London, UNITED KINGDOM

## Abstract

Heterosexual transmission of HIV-1 is characterized by a genetic bottleneck that selects a single viral variant, the transmitted/founder (TF), during most transmission events. To assess viral characteristics influencing HIV-1 transmission, we sequenced 167 near full-length viral genomes and generated 40 infectious molecular clones (IMC) including TF variants and multiple non-transmitted (NT) HIV-1 subtype C variants from six linked heterosexual transmission pairs near the time of transmission. Consensus-like genomes sensitive to donor antibodies were selected for during transmission in these six transmission pairs. However, TF variants did not demonstrate increased viral fitness in terms of particle infectivity or viral replicative capacity in activated peripheral blood mononuclear cells (PBMC) and monocyte-derived dendritic cells (MDDC). In addition, resistance of the TF variant to the antiviral effects of interferon-α (IFN-α) was not significantly different from that of non-transmitted variants from the same transmission pair. Thus neither *in vitro* viral replicative capacity nor IFN-α resistance discriminated the transmission potential of viruses in the quasispecies of these chronically infected individuals. However, our findings support the hypothesis that within-host evolution of HIV-1 in response to adaptive immune responses reduces viral transmission potential.

## Author Summary

Despite the available HIV-1 diversity present in a chronically infected individual, single viral variants are transmitted in 80–90% of heterosexual transmission events. These breakthrough viruses may have unique properties that confer a higher capacity to transmit. Determining these properties could help inform the rational design of vaccines and enhance our understanding of viral transmission. We isolated the transmitted variant and a set of related non-transmitted variants from the transmitting partner near the estimated date of transmission from six epidemiologically linked transmission pairs to investigate viral correlates of transmission. The simplest explanation that transmitted variants are inherently more infectious or faster replicators *in vitro* did not hold true. In addition, transmitted variants did not replicate more efficiently than their non-transmitted counterparts in dendritic cells or in the presence of interferon-alpha *in vitro*, suggesting that they are not uniquely adapted to these components of the innate immune system. More ancestral genomes that were relatively sensitive to antibody neutralization tended to transmit, supporting previous reports that mutational escape away from the adaptive immune response likely reduces the ability to transmit. Our investigation into the traits of transmitted HIV-1 variants adds to the understanding of viral determinants of transmission.

## Introduction

HIV-1 transmission is characterized by an extreme genetic bottleneck, the basis of which is unclear. Studies of both the highly diverse envelope glycoprotein [1–3] and full HIV-1 genomes [4] demonstrated that 80–90% of heterosexual transmissions are initiated by a single virus variant selected from the diverse viral quasispecies present in the chronically infected transmitting partner. These variants, which are different in each transmission event, have been named transmitted/founder (TF) viruses. Studying TF viruses could enhance our understanding of viral transmission and inform HIV prevention strategies.

The TF is rarely the dominant variant in the plasma or genital tract of the transmitting partner [5,6], which suggests that transmission is not entirely stochastic and may involve selection. A number of prior studies have identified distinctive properties of TF variants [4,7–19], particularly in analyses of the TF viral envelope (Env) glycoprotein. Reported characteristics of TF virus Envs include a selection for CCR5-tropism [2,20], a predominance of shorter and less glycosylated Env proteins [1,11,15,18,19], a preference for binding α4β7 [10,21] and a selection for more ancestral variants [8,22]. Although these studies observed selection of viral traits, others found that acute and chronic variants had similar characteristics. By generating infectious molecular clones (IMC) with the *env* genes from linked recipients and transmitting partners in a common viral backbone, acute and chronic donor viruses displayed similar CD4 and CCR5 requirements for cell entry, low macrophage tropism, and no preferential usage of alternative coreceptors [23,24]. Furthermore, studies of *env* only clones from acute infection compared with chronic control viruses have shown similar CD4 T cell subset tropism, low macrophage tropism, and a lack of effect of blocking α4β7 on infection [25].

Selection of viral traits outside of the *env* gene has also been observed during heterosexual transmission. We recently described a selection bias during transmission for more consensus-like HIV-1 variants, in *gag*, *pol* and *nef* genes, from a cohort of 137 subtype C infected epidemiologically-linked transmission pairs [7]. This study suggested that *in vivo* fitness of consensus-like HIV-1 variants increased their likelihood of transmission [7]. Studies of full-length infectious molecular clones of TF viruses, in comparison to control viruses derived from chronic infection, have also demonstrated increased particle infectivity, as well as an enhanced resistance to interferon-α (IFN-α) in TF viruses [13,17].

While informative, conclusions of these previous studies are limited in that only individual genes were examined, or corresponding non-transmitted (NT) variants from the transmitting partner were unavailable as controls. HIV-1 IMC with the full complement of HIV-1 proteins have not been generated from both partners of transmission pairs nor evaluated for genetic and phenotypic signatures during transmission. Characterizing TF variants in comparison to NT variants from epidemiologically-linked partners could provide further insight into the viral requirements of HIV-1 transmission, potentially leading to new targets for intervention.

Here, we describe genetic and phenotypic comparisons of full-length genome TF and NT variants from six subtype C epidemiologically-linked heterosexual transmission pairs. We amplified and sequenced near full-length HIV-1 genomes by single genome amplification (SGA) to assess genetic selection during transmission. In addition, we cloned the complete TF genome along with a representative panel of NT variants. These clones were used to assess the relative *in vitro* fitness of TF variants as measured by particle infectivity, neutralizing antibody resistance, replicative capacity in PBMC and dendritic cells, as well as IFN-α resistance. We found a strong selection bias toward consensus sites across the entire genome, at both the amino acid and nucleotide level, in all six pairs. The TF variants were also more sensitive to neutralization by donor antibodies than NT variants. However, no evidence was found for TF variants exhibiting increased particle infectivity, replicative capacity, or IFN-α resistance when compared to the transmitting partner’s NT variants. Thus, in these six subtype C transmission pairs the transmission potential of TF variants is not discriminated by inherent *in vitro* replicative capacity or interferon resistance, and may be determined by alternate phenotypes difficult to dissect in these *in vitro* systems.

## Results

### Transmission pairs and amplification of near full-length genomes

Full-length genome HIV-1 variants derived from linked transmission pairs have yet to be evaluated for characteristics associated with transmission. To define whether TF variants exhibit distinct properties, we compared them to their NT counterparts in six heterosexual epidemiologically-linked transmission pairs. We selected five female-to-male and one male-to-female therapy-naïve subtype C epidemiologically-linked transmission pairs from the Zambia-Emory HIV Research Project (ZEHRP) based on the availability of plasma samples at the nearest time points following transmission (average 28 days post estimated date of infection) (Table 1). We PCR amplified, using a high-fidelity polymerase, and sequenced a total of 167 HIV-1 near full-length single genome amplicons as described previously [26]. All six linked recipients were in Fiebig Stage II of infection, and were infected with a single variant from the donor quasispecies, as demonstrated by star-like phylogeny in a median of 8 near full-length genome amplicons per sample [2,4]. This allowed us to infer an unambiguous consensus TF sequence from the genetically homogeneous population of sequences in each linked recipient.

**Table 1:** Transmission pair characteristics.

Coded ID^a^	Partner Status^b^	EDI^c^	Sample Date^d^	Days after EDI	VL^e^	VL Sample Date	# Sequences Analyzed
Z331M	LR	17-Mar-09	18-Apr-09	32	2,864,668	18-Apr-09	9
Z331F	D		15-Apr-09	29	2,620	2-May-09	20
Z3576F	LR	6-Mar-09	28-Mar-09	22	6,460,200	28-Mar-09	18
Z3576M	D		18-Apr-09	43	54,100	23-Apr-09	15
Z3618M	LR	27-May-09	18-Jun-09	22	16,600,000	18-Jun-09	8
Z3618F	D		11-Jul-09	45	20,400	15-Jul-09	17
Z3678M	LR	26-Aug-09	17-Sep-09	22	3,017,616	30-Sep-09	8
Z3678F	D		23-Sep-09	28	269,240	23-Sep-09	18
Z4248M	LR	13-May-10	4-Jun-10	22	24,993,584	4-Jun-10	6
Z4248F	D		11-Jun-10	29	119,320	11-Jun-10	21
Z4473M	LR	16-May-11	7-Jun-11	22	16,891,328	7-Jun-11	9
Z4473F	D		7-Jun-11	22	104,427	18-Jun-11	18

^a^ Z = Zambia, M = male, F = female

^b^ LR—epidemiologically linked recipient partner, D—donor partner.

^c^ EDI—estimated date of infection (22 days prior to Ag+Ab- sample) [3]

^d^ Sample Date—date of collection of plasma from which viral RNA was extracted

^e^ VL—Viral Load (RNA copies/mL)

**Fig 1:**
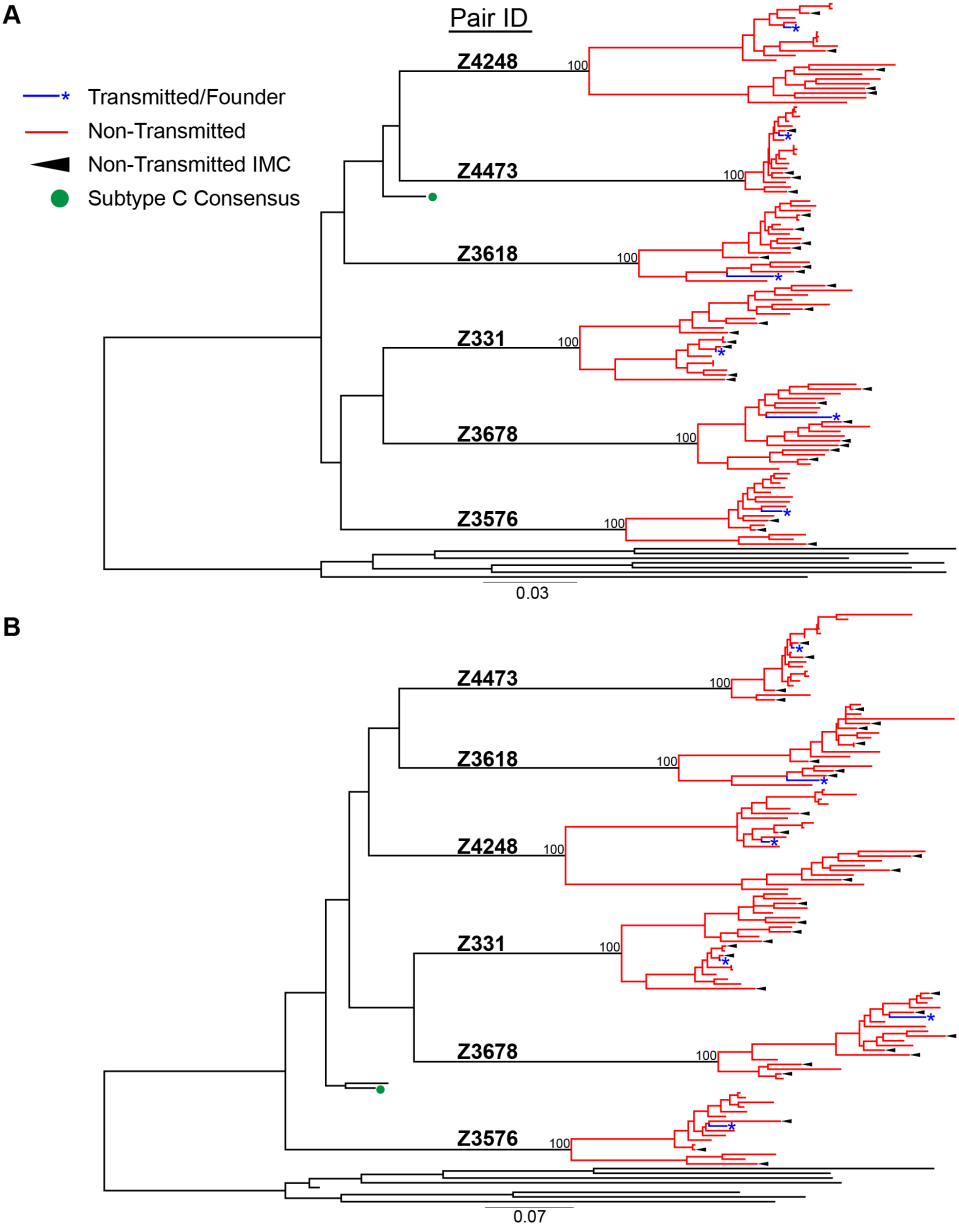
HIV-1 Full-length genome phylogenetic analysis of six epidemiologically-linked heterosexual transmission pairs. (A) Nucleotide sequences for all 115 single genomes amplified from six linked transmission pairs were aligned to the curated LANL consensus/ancestral alignment and a maximum likelihood tree was generated. (B) Single genome nucleotide sequences for each viral gene (*gag*, *pol*, *vif*, *vpr*, *vpu*, *tat*, *rev*, *env*, & *nef*) were translated to their amino acids and then concatenated. These were aligned with LANL consensus/ancestral concatenated protein sequences and a maximum likelihood tree was generated. Transmitted/founder sequences from linked recipients are in blue, donor non-transmitted variants are in red, LANL database curated consensus/ancestral sequences are shown in black, where the LANL subtype C consensus is indicated by a green circle. Black arrows indicate virus variants from the donor quasispecies that were selected for generation of non-transmitted (NT) infectious molecular clones.

For phylogenetic analyses, we aligned full-length nucleotide sequences as well as concatenated full proteome amino acid sequences of 115 HIV-1 single genomes (each TF virus represented by a single consensus sequence), with the HIV-1 consensus/ancestral alignment from the Los Alamos National Laboratory (LANL) HIV database. We generated maximum likelihood trees of the full-length genome and proteome alignments for all six transmission pairs, and confirmed that all pairs were epidemiologically linked, since each TF variant fell clearly within the branches of the linked donor virus variants. Each transmission pair clustered independently on the phylogenetic tree with bootstrap values of 100 (Fig 1). All six linked donor partners were chronically infected and demonstrated viral diversity in their plasma near the time of transmission (Fig 1).

### Consensus-like nature of TF and NT viruses

**Fig 2:**
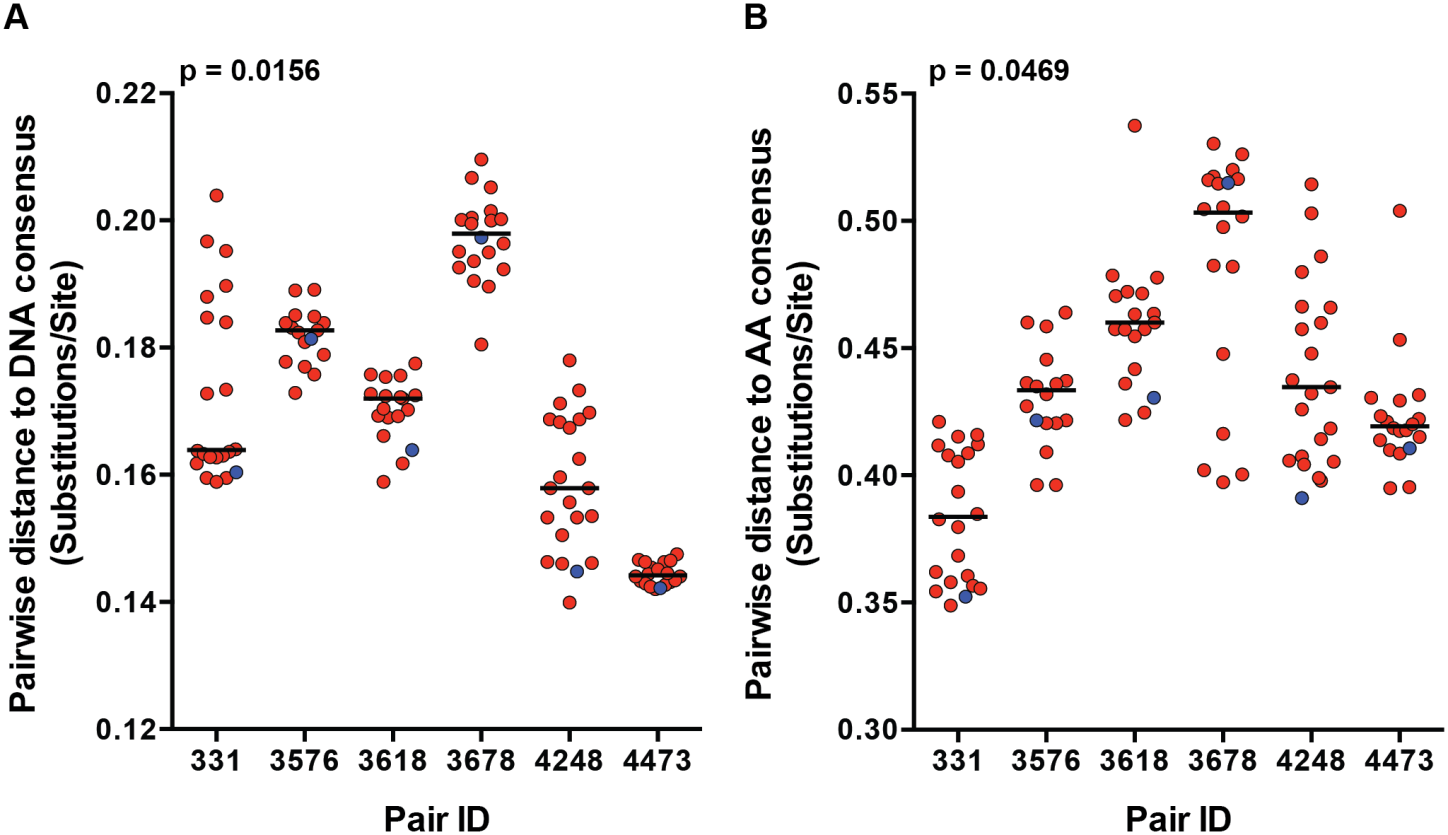
Transmission selects for more consensus-like TF variants. The pairwise distance of each viral variant on the (A) nucleotide and (B) amino acid phylogenetic trees to the LANL subtype C consensus node were measured and plotted for each transmission pair. Transmitted/founder variants are in blue, and non-transmitted variants are in red. The median of the non-transmitted variants is designated with a black line. The statistical significance of the difference between TF and NT donor median values was analyzed using a one-tailed Wilcoxon matched-pairs signed rank test.

We previously demonstrated a consistent transmission bias for variants with consensus-like amino acid residues across the Gag, Pol and Nef proteins by population sequencing in a cohort of 137 epidemiologically-linked subtype C transmission pairs [7]. Although this finding has been shown for the *gag* and *env* genes independently, it has not been confirmed by full-length genome SGA from the transmitting partner's quasispecies [7,8]. We examined the selection bias for more consensus-like viruses by measuring the pairwise distance (branch length), of each viral variant to the LANL subtype C consensus node on the full-length nucleotide and amino acid phylogenetic trees (Fig 1). TF variants had a significantly shorter pairwise distance to the subtype C consensus node than the median of their corresponding NT variants for both nucleotide (Fig 2A; p = 0.0156) and amino acid (Fig 2B; p = 0.0469) sequences. These transmission pairs confirm, as previously described, a selection bias for consensus-like amino acid and nucleotide sites across the viral genome during transmission.

### Particle infectivity of TF and NT viruses

In a previous study, TF virions exhibited enhanced infectivity in comparison to chronic control viruses on TZM-bl cells [13]. To test particle infectivity within transmission pairs, we generated full-length IMC for 40 viral variants, including the 6 TF variants and 3–8 NT variants from each chronically infected transmitting partner, as described previously [26]. We selected variants to represent the genetic diversity present in the donor near the time of transmission (Fig 1), and confirmed that the IMC and amplicon sequences were identical by whole genome sequencing. We also excluded the rare sequences that contained gross genetic defects, such as large deletions and frameshift mutations in gene coding regions. For each IMC, we generated virus stocks by transfection of 293T cells.

**Fig 3:**
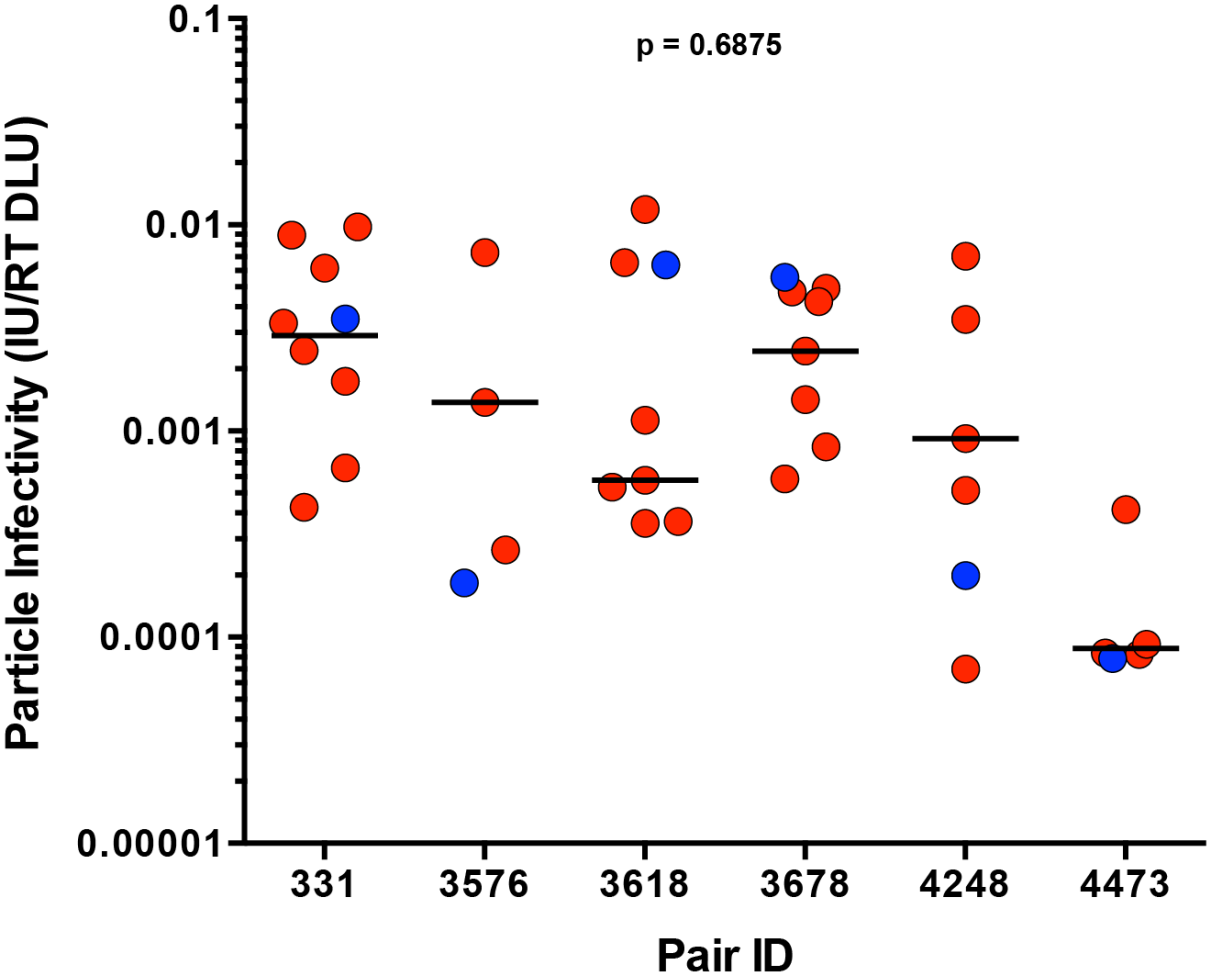
Particle infectivity of TF and NT infectious molecular clones. 293T cells were transfected with TF (blue) and NT (red) infectious molecular clones. Supernatants collected 48 hours post-transfection were titered on TZM-bl cells to define the number infectious units per microliter (IU/ul), while reverse transcriptase activity was measured simultaneously for total viral particles per microliter (RT DLU, reverse transcriptase digital light units). The particle infectivity (IU/RT DLU) of each infectious molecular clone is plotted for each transmission pair. The median of the NT variants is designated with a black line. The statistical significance of the difference between TF and NT donor median values was determined using a two-tailed Wilcoxon matched-pairs signed rank test (p = 0.6875).

We defined particle infectivity as the ratio of infectious units, as measured by the virus titer on TZM-bl cells, a standard reporter cell line whose permissivity correlates with that of PBMC [27], over total amount of virions, measured by reverse transcriptase activity of the virus stock. We confirmed that the particle infectivity of a subset of virus stocks generated from 293T cells and harvested 48 hours after transfection (for consistency, as particle infectivity decreased over time post-transfection, S1A Fig) correlated with the particle infectivity of virus stocks generated from PBMC 8 days following infection (S1B Fig, p < 0.0001, r = 0.9455). Analysis of the particle infectivity of virus stocks produced from all of the infectious molecular clones showed that the particle infectivities of all viruses tested ranged from 7x10^-5^ to 1x10^-2^, and that there was also a wide range of particle infectivities within each transmitting partner’s quasispecies (Fig 3). In pair 3678, the TF variant was the most infectious virus compared to the rest of the transmitting partner’s variants, while the TF from pair 3576 was the least infectious (Fig 3). TF variants spanned the thousand-fold range of particle infectivities measured for all the viruses tested, as can be seen by the TF from pairs 3618 and 4473, which are found on extreme ends of the particle infectivity spectrum. Across all six transmission pairs, we observed no significant selection for infectivity when comparing the TF to the median of the transmitting partner’s quasispecies (Fig 3; p = 0.6875). In these subtype C transmission pairs particle infectivity did not constitute a dominant determinant of transmission fitness.

### Sensitivity of TF and NT variants to neutralization by antibodies present in the transmitting partner

**Fig 4:**
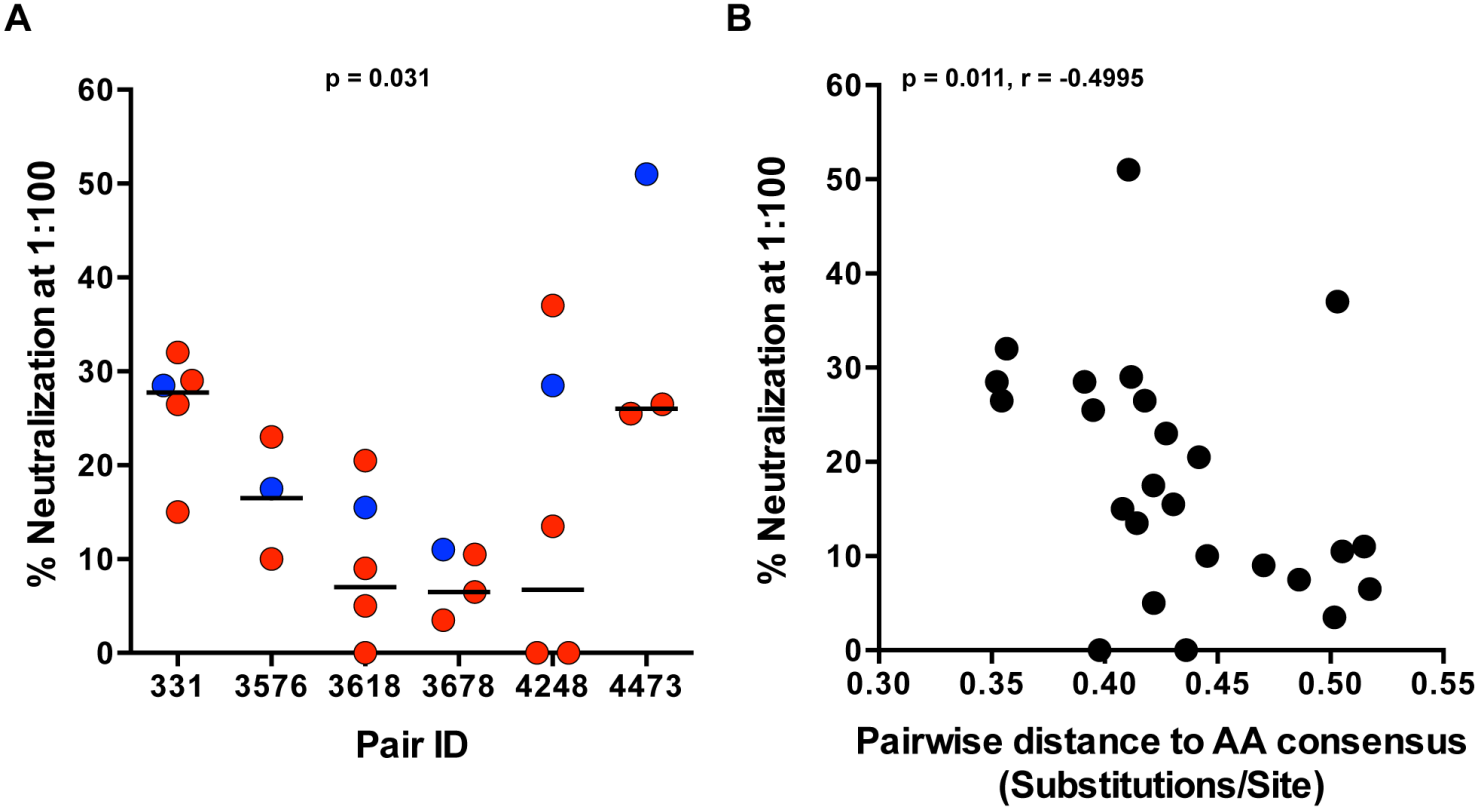
TF variants are more sensitive to neutralization by donor plasma than NT viruses. (A) Neutralization of TF (blue) and NT (red) IMC by donor plasma was measured for each pair in a TZM-bl neutralization assay. Percent neutralization by donor plasma (diluted 1:100) is depicted on the y-axis, and representative TF and NT viruses tested for each transmission pair are depicted on the x-axis. The median of the NT variants is designated with a black line. The statistical significance of the difference between TF and NT donor median values was determined using a two-tailed Wilcoxon matched-pairs signed rank test (p = 0.031). (B) Spearman correlation of the pairwise distance to the amino acid subtype C consensus and donor plasma neutralization described in part A over all the variants tested from 6 transmission pairs (p = 0.011, r = -0.4995).

We previously reported that Env glycoproteins derived from early viruses in acutely infected linked recipients were on average more sensitive to neutralization by plasma from the transmitting partner, compared to autologous Envs directly derived from the transmitting partner [1]. Antibody neutralization of SGA-derived genome length TF and NT variants, derived from the first month of infection, from heterosexual epidemiologically-linked transmission pairs, has not been examined to date. Using a previously described TZM-bl neutralization assay [1,28], we evaluated neutralization of full-length TF and autologous NT IMC by plasma from the transmitting partner near the time of transmission. Donor plasma (diluted 1:100) demonstrated relatively weak neutralization against the majority of viruses tested in each panel, with a median of 18% neutralization. The highest level of neutralization was seen in pair 4473 against the TF (51%) (Fig 4A). Overall, TF variants were more efficiently neutralized compared to the medians of the transmitting partner’s NT variants (Fig 4A; p = 0.031). Additionally, greater neutralization negatively correlated with distance to the amino acid subtype C consensus (Fig 4B; p = 0.011, r = -0.4995), suggesting a link between these two measurements. Consistent with our previous findings, transmission did not select for TF variants with greater neutralizing antibody resistance to donor plasma.

### *In vitro* replication of TF and NT viruses

**Fig 5:**
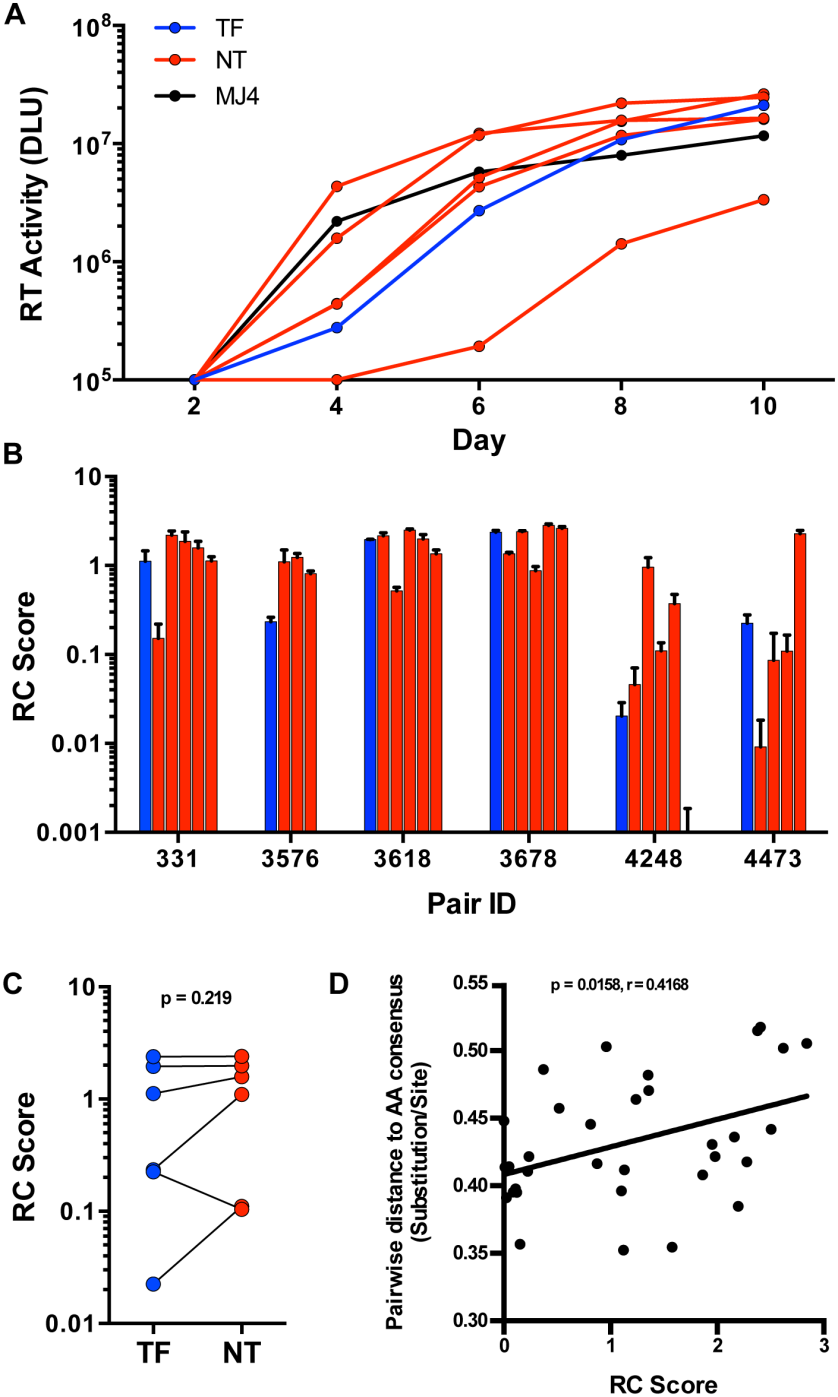
*In vitro* replication of TF and NT viruses in PBMC. (A) Virus growth over 10 days in PBMC culture as measured by reverse transcriptase (RT) activity (DLU = digital light units) of the TF (blue), NT variants (red) and MJ4 standard (black) for one representative transmission pair, 331. (B) Replicative capacity (RC) scores, based on the area under the curve relative to MJ4, of all tested TF (blue) and NT (red) variants from six transmission pairs. (C) RC scores of the TF (blue) compared to the median of the corresponding NT variants (red) (Wilcoxon matched-pairs signed rank test, two-tailed p = 0.219). (D) Spearman correlation of the pairwise distance to the amino acid subtype C consensus described in Fig 2 and RC scores over all variants tested (Non-parametric Spearman p = 0.0158, r = 0.4168.) The linear regression line is shown for visualization purposes.

Selection for consensus-like TF variants in these six transmission pairs may indicate a selection for viruses with greater *in vivo* fitness, as hypothesized from a study of 137 linked transmission pairs [7]. To determine whether this translated into a similar fitness advantage in activated CD4 T cells, we measured the replicative capacity (RC) of viruses *in vitro*. TF and NT IMC were tested for *in vitro* replication by infection of stimulated peripheral blood mononuclear cells (PBMC), at equal multiplicities of infection (MOI). Since the number of infectious particles to total particles varied greatly between all virus stocks tested, we based the amount of virus used for each replication experiment on a consistent MOI (0.01), rather than equal amounts of virus particles, in order to normalize for initial infectivity. We measured virus growth by reverse transcriptase activity of cell culture supernatants every 48 hours for ten days (Fig 5A). RC scores were generated for each virus based on the area under the curve of virus growth, as described in the methods. TF viruses exhibited a wide range of RC among all the viruses tested, and the relative RC of TF as compared to NT viruses from the same donor also varied substantially (Fig 5B). For instance, the TF from pair 3576 had the lowest RC when compared to the transmitting partner’s quasispecies. Alternatively, pairs 3618 and 3678 had TF viruses with relatively high RC, although they were not the highest replicators from their transmitting partner’s quasispecies (Fig 5B). In total, we saw no significant selection for TF viruses having higher *in vitro* RC than the median RC of the NT viruses tested (Fig 5C; p = 0.219). Similar to particle infectivity, which correlated with RC over all the viruses tested (S2 Fig; p = 0.0005, r = 0.5712), there was no evidence for a distinct replicative capacity profile associated with transmission. In addition, viruses closer to consensus typically had lower *in vitro* replicative capacities, since the pairwise distance to subtype C consensus correlated with *in vitro* replicative capacity (Fig 5D; p = 0.0158, r = 0.4168).

Since dendritic cells have also been implicated as an initial target cell for establishment of HIV-1 infection in the genital mucosa [29], we examined the ability of the 6 TF and a limited set of 6 NT variants with similar *in vitro* RC scores, to productively infect and replicate in immature monocyte derived dendritic cells (MDDC) *in vitro*. We cultured MDDC by isolation and differentiation of blood-derived CD14+ monocytes from healthy donors and infected them with virus at a high MOI of 1. We assayed virus production by measuring the reverse transcriptase activity present in cell culture supernatants every 48 hours for twelve days. We found that the TF and NT variants studied did not significantly differ in their ability to replicate in MDDC (S3 Fig; p = 0.87). Of the twelve TF & NT variants, six had detectable replication in MDDC (3 TF & 3 NT), suggesting that productive infection of MDDC is limited, even at a high MOI, and is not a requirement for transmission. Overall, these data suggest that HIV-1 transmission is permissive to TF variants with a wide range of *in vitro* replicative capacities relative to the transmitting partner’s quasispecies.

### Interferon-α resistance in HIV-1 subtype C transmission

By conducting *in vitro* replication assays in cells pre-treated with exogenous interferon-α (IFN-α), previous studies found that subtype B and subtype C TF variants were relatively resistant to IFN-α compared to a panel of chronic viruses [13] or later variants from the same individual [17]. These studies suggested a selection during the HIV-1 transmission bottleneck for variants adept at escaping innate immunity, specifically the antiviral effects of IFN-α. However, these studies were not done in epidemiologically-linked transmission pairs, and thus were unable to directly compare TF viruses to related NT variants in the donor quasispecies near the time of transmission. To test whether the subtype C TF viruses investigated here exhibited relative resistance to IFN-α, as compared to NT variants derived from the transmitting partner's quasispecies, we assayed *in vitro* virus replication in PBMC in the presence and absence of IFN-α.

**Fig 6:**
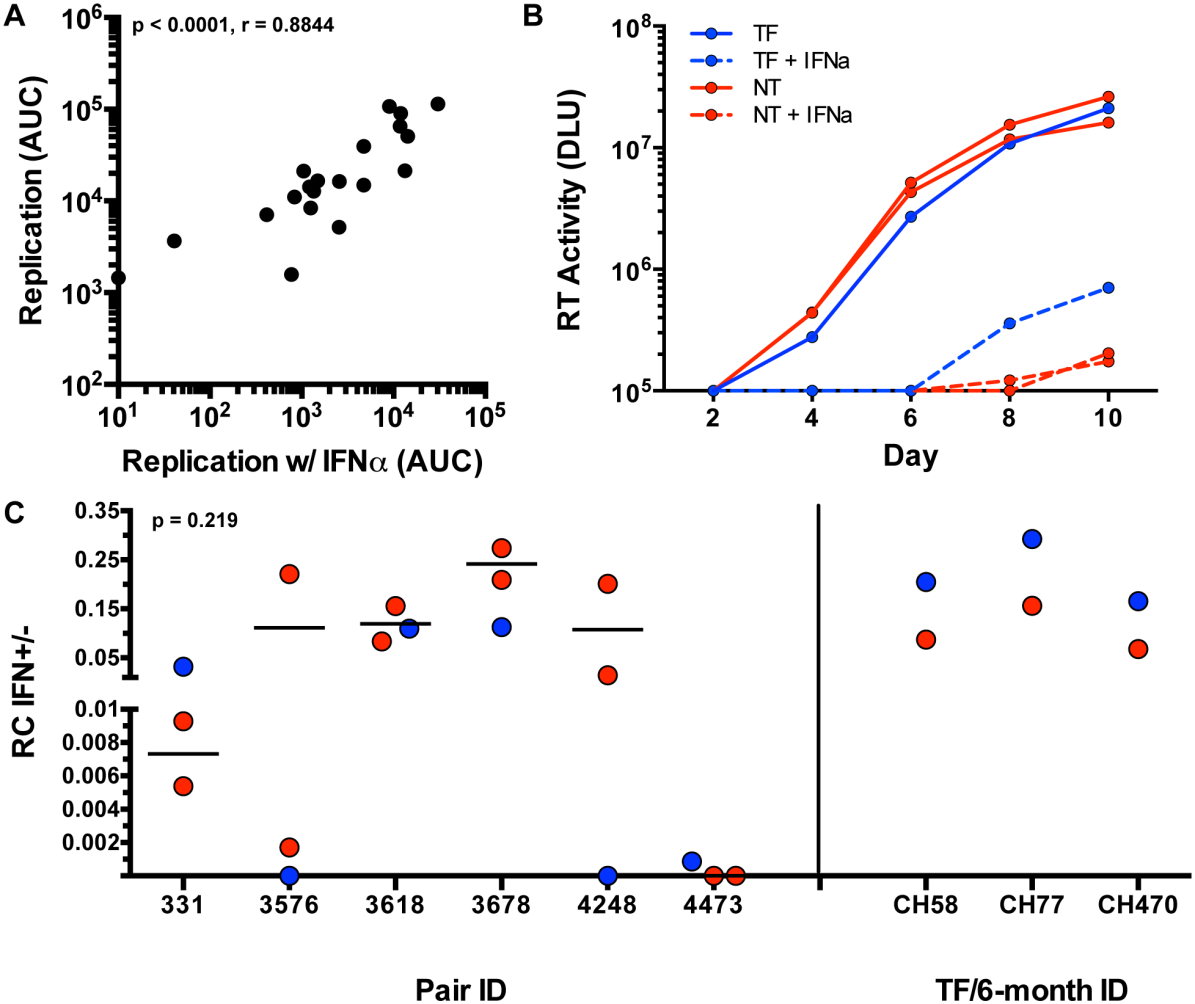
Interferon-α resistance of TF and NT viruses. (A) Spearman correlation of the replication measured by area under the curve (AUC) (y-axis) of each tested variant (black dots) and the replication (AUC) in the presence of interferon alpha (x-axis) (Non-parametric Spearman p < .0001, r = 0.8844). (B) Virus growth over 10 days in culture as measured by reverse transcriptase (RT) activity (DLU = digital light units) of the TF (blue), and NT variants (red) in the presence of IFN-α (dotted) and absence of IFN-α (solid lines) in an example pair 331. (C) RC scores in the presence of IFN-α were divided by the RC score in the absence of IFN-α for TF (blue) and selected NT (red) viruses with similar replication kinetics (Wilcoxon matched-pairs signed rank test, two tailed, p = 0.219). Subtype B TF (blue) and 6-month consensus (red) viruses are shown as controls on the right.

We assayed viral replication in activated CD8-depleted PBMC in the presence and absence of 5,000 U/ml of IFN-α, which was added 24 hours prior to infection in order to maximally inhibit viral replication, as described previously [17]. Supernatant HIV-1 p24 antigen levels were measured every 48 hours for 10 days to assess the kinetics of viral replication. In the initial 21 variants tested, growth of virus in the presence of IFN-α was tightly correlated with *in vitro* RC scores in the absence of IFN-α (Fig 6A; p < 0.0001, r = 0.8844), suggesting that *in vitro* growth in the presence of IFN-α was largely determined by viral replicative capacity.

In light of this, we attempted to delineate subtle differences in IFN-α resistance by performing further experiments with selected NT variants that exhibited relatively similar replication kinetics to the TF in each pair (to minimize the impact of replication differences). The replication of these selected viruses was assessed in activated PBMC in the presence and absence of 1,000 U/ml of IFN-α (added 24 hours prior to infection), monitoring virus replication by reverse transcriptase activity in the supernatant. An example of such an assay for transmission pair 331 is shown in Fig 6B. When compared to the tested NT variants, TF viruses did not differ significantly in resistance to IFN-α (assessed as the ratio of the RC score in the presence and absence of IFN-α) (Fig 6C, p = 0.219). In pair 331 and 4473, the TF appeared to be more IFN resistant than the NT viruses from the same donor (Fig 6C). In pair 3618 the TF was near the median of the NT variants, while in three pairs (3576, 3678, and 4248), the TF was the most sensitive to IFN-α. Overall, the IFN-α resistance of the TF viruses did not differ significantly from the median of the NT variants (Fig 6C).

Because the TF viruses were not found to be more IFN-resistant than donor NT viruses, we validated the method used for analysis of IFN resistance with 3 subtype B TF and 6-month consensus virus pairs that had previously been demonstrated to differ in their IFN-α resistance [17]. As shown in Fig 6C, the 3 TF viruses were each confirmed to be more IFN-resistant than the matched 6-month virus from the same subject, verifying the ability of the methods used here to detect previously documented differences in viral IFN resistance. In the six subtype C epidemiologically-linked transmission pairs studied we also observed that IFN-α resistance correlated with the virus' ability to replicate (S4A Fig). Although the RC and IFN-α resistance of the six subtype B TF and 6-month viruses was not statistically correlated, these subtype B TF viruses did have higher RC scores than their matched 6-month variants (S5 Fig). Overall, these data suggest that a component of IFN-α resistance is the ability of TF and NT HIV-1 variants to replicate.

To confirm that this finding was independent of the amount of IFN used to inhibit viral growth, we measured replication at day 7 for four viruses with a representative range of RC scores using a range of IFN-α concentrations (0.5 U/ml–10,000 U/ml). The relative sensitivity of these viruses was consistent across the range of IFN-α concentrations tested (S4B Fig). Additionally, we tested a limited subset of TF and NT variants for their ability to induce IFN-α, which may have influenced IFN-α resistance measurements, and found that IFN-α levels above background were not detectable at day 8 in either PBMC or MDDC infected cultures (S4C Fig). Hence HIV-1 transmission from these donors was not mediated by TF viruses that exhibited higher levels of interferon resistance than NT viruses, indicating that heterosexual HIV-1 transmission is permissive to viruses anywhere within the range of *in vitro* interferon resistance profiles observed in the donors studied here, and factors other than IFN-α resistance constituted the dominant determinants of transmission fitness in these pairs.

## Discussion

The rapid within-host diversification of HIV-1 observed during chronic infection, which represents a primary obstacle to effective HIV prevention strategies, contrasts starkly with the viral homogeneity evident following transmission. The stringent genetic bottleneck is most pronounced in heterosexual transmission, where a vast majority of new infections are established by single viral variants. Correlates of transmission may become evident by studying the properties of these transmitted/founder (TF) variants, which, in turn, could help inform effective HIV-1 vaccine design. Studies of early and transmitted variants have found genetic and phenotypic signatures associated with transmission; however, none have examined full-length TF variants and corresponding non-transmitted (NT) variants present near the time of transmission from epidemiologically-linked transmission pairs.

In this study, we applied new molecular techniques to investigate the requirements of HIV-1 transmission in six subtype C transmission pairs. We amplified, sequenced, and generated infectious molecular clones (IMC) of matched full-length TF viruses very early after infection (Fiebig stage II) and near full-length NT variants from 22–45 days following the estimated date of transmission. Technical limitations associated with amplifying full-length virus from genital tract samples required us to amplify from patient plasma. Despite this limitation, we previously showed in eight epidemiologically-linked transmission pairs that the TF was most highly related to NT variants that were absent from the predominant genital tract subpopulations, and were found in both blood and genital tract of the donor partner [5]. Consistent with this, in pair 331 we observed a NT variant in the plasma of the transmitting partner with only three amino acid differences from the TF across the entire proteome (Fig 1B).

In this study, we generated IMCs from the diverse donor quasispecies with great sequence accuracy and selected variants in an unbiased fashion. Five of the six pairs were female-to-male, the route by which the most stringent bottleneck occurs [7,30]. Since high donor viral load and the presence of genital ulcers and inflammation (GUI) in the recipient can, to a certain degree, mitigate selection bias in the bottleneck, it is important to note that these six pairs include three donors with viral loads >100,000 RNA copies/ml, as well as one recipient with a reported GUI in the twelve months prior to seroconversion [3,7]. Despite these caveats, single variant transmission was observed in all six pairs.

Consistent with our previous findings [7], we observed selection during transmission for variants with more consensus-like amino acid and nucleotide DNA sequences from the available quasispecies present in the donor at the time of transmission, across the full viral proteome and genome, respectively (Fig 2). It has been shown that HIV-1 within-host diversity during chronic infection is greater than between-host diversity, suggesting conservation of certain genetic elements during transmission [31]. In conjunction, studies of subtype A and D heterosexual transmission pairs demonstrated transmission of more ancestral viral variants, by measuring distances of each variant to their most recent common ancestor (MRCA) on a phylogenetic tree of Env sequences [8,22]. In the current study, the LANL subtype C consensus node falls near the subtype C MRCA highlighting the equivalence of these two measurements (Fig 1). Thus, HIV-1 transmission consistently selects for variants that more closely resemble ancestral and consensus-like viruses, indicating that evolution in the host decreases transmission potential.

The viral diversification observed during chronic infection due to adaptive immune pressure targeted specifically against HIV-1 is likely driving viral evolution away from consensus [1,7,32,33]. We have previously shown that acquisition of resistance to antibody neutralization comes with a transmission fitness cost [1]. We similarly found that TF viruses were more sensitive to neutralization by donor plasma acquired near the time of transmission when compared to the corresponding NT variants. It should be noted, however, that a limitation to this finding is that NT variants were cloned and tested with plasma from approximately four weeks after the estimated date of infection, although the two pairs with the largest time gap between transmission and sampling did not show the greatest neutralization of the TF. These data also reaffirm that TF variants are generally not resistant to antibody neutralization [1,34]. As expected, donor plasma tested against contemporaneous viruses (TF or NT) demonstrated limited neutralization capacity. Moreover, neutralization sensitivity correlated with the distance to consensus over all the viruses tested. Considering these observations, it is reasonable to propose that selection of antibody sensitive variants during transmission is a side effect of the transmission cost associated with non-consensus adaptations in general, and not an underlying mechanism of transmission itself.

In order to address the role of viral fitness in transmission, we measured the *in vitro* fitness of a subset of viruses from six transmission pairs. Although a previous study found that TF viruses were more infectious than chronic control viruses [13], we found no bias towards increased infectivity when comparing the TF to the corresponding NT variants. Particle infectivity in TZM-bl cells correlated with replicative capacity in PBMC, suggesting that entry into TZM-bl cells is representative of a component of viral replication in primary cells. Viral replicative capacity in activated PBMC, a fundamental measure of *in vitro* fitness, was also not higher for TF variants in comparison to the corresponding NT variants, and none of the TF variants exhibited the highest replicative capacity from among the tested NT variants. We found that more consensus-like variants, which are typically those that transmit, had lower *in vitro* replicative capacities over all the variants tested, indicating that higher *in vitro* replicative capacity is not linked to transmission. TF variants were also not observed to have enhanced replicative capacity in monocyte-derived dendritic cells, an *in vitro* model for dendritic cells, which may act as an initial target cell for establishment of HIV-1 infection. These findings argue against the original hypothesis that consensus-like variants would have higher *in vitro* replicative capacities. Thus, *in vitro* RC in activated PBMC or MDDC may not reflect *in vivo* transmission fitness, potentially because replication in stimulated PBMC may recapitulate the inflammatory environment that occurs some time after transmission and during chronic infection rather than conditions initially encountered at initial sites of virus replication. We cannot rule out the possibility that replication assays in cell types more representative of mucosal transmission, such as tissue resident CD4+ T cells or Langerhans cells, may yield different results. However, consistent with our observations, previous studies found a significant negative correlation between similarity to consensus and *in vitro* RC in a larger number of patients in differing cohorts using gag-chimeras [35,36]. Transmission of low *in vitro* fitness variants may seem counterintuitive; however, full-length TF IMC as well as over 200 transmitted Gag chimeras have been shown to exhibit a wide range of *in vitro* replicative capacities [35,37,38], as we found for our six TF viruses. A recent theoretical model of HIV transmission predicted that variants with lower replicative capacity via increased latency would exhibit a greater transmission potential *in vivo* [39], and it is therefore possible that modestly lower *in vitro* replicative capacity is an advantage during transmission.

A potential selection factor during mucosal transmission is the early innate immune response to HIV-1. Innate antiviral cytokines including IFN-α are induced at initial sites of HIV-1 replication in the mucosa and draining lymph nodes [40,41], hence HIV-1 variants that are more resistant to the antiviral effects of IFN-α may have an advantage during transmission. Indeed, cross-species transmission of Simian Immunodeficiency Virus (SIV) to humans required escape from the interferon stimulated APOBEC3 restriction factors by enhanced Vif antagonism [42]. A recent in-depth study using the rhesus macaque model also found that IFN-α treatment prior to intrarectal SIV_MAC251_ inoculation reduced the number of transmitted variants and increased the number of challenges necessary to initiate infection [43]. Consistent with the hypothesis that type 1 IFNs contribute to the transmission bottleneck, previous studies using HIV-1 found that TF variants are generally more resistant to IFN-α *in vitro* than viruses present during early chronic infection [13,17]. Fenton-May et. al. [17] found that TF viruses from both subtype B and C infected subjects were more resistant to IFN-α when compared to matched variants generated from the same individual six months post-infection or during early chronic infection. Parrish et. al. [13] found that TF viruses are more resistant to IFN-α than viruses from unmatched chronic controls, though this was true only for the subtype B and not for the subtype C variants they studied. In six subtype C transmission pairs studied here we did not observe that TF viruses exhibited enhanced resistance to IFN-α compared to NT viruses. TF variants did not replicate to higher levels in the presence of IFN-α, nor did they have higher ratios of replication in the presence versus the absence of IFN-α.

These differing results could be due to differences in experimental protocols, as well as difficulties in separating inherent replicative capacity from interferon resistance. We therefore tested the IFN-α resistance of previously studied TF and 6-month viruses and confirmed that these TF variants were more resistant to the effects of IFN-α, consistent with previous observations. In addition, we found that the TF variants had higher replicative capacities than the 6-month consensus variants, although for this group of viruses IFN-α resistance did not directly correlate with viral replicative capacity. The influence of viral replicative capacity on measures of interferon resistance is not fully understood. The impact of multiplicity of infection on measured interferon resistance has been noted previously [44], so in the current studies we utilized a low multiplicity to ensure adequate target cell availability even for the higher replicating viruses. We chose a MOI of 0.01 for our assays since it represented an input virus dose at which we were able to measure both replication differences between viruses, as well as IFN-α resistance differences (S4D Fig). For the viruses tested from the six Zambian transmission pairs, we found that *in vitro* replication in the presence of interferon correlated with replication in the absence of IFN-α, such that the rank order of virus replicative capacities from lowest to highest was similar in the presence or absence of interferon. Even when a subset of viruses with more closely-matched levels of replication were studied so that we were better able to observe IFN-α resistance differences, we found that TF variants were not IFN-α resistant compared to the matched NT variants.

The lack of difference in the IFN resistance of TF and NT viruses in these transmission pairs may be due to the length of time for which the chronically infected viral donors had been infected prior to viral transmission to their partners and derivation of the viruses studied. Fenton-May et. al. showed that while IFN-α resistance decreased over the first 6 months following infection, it subsequently increased in different subjects at timepoints from 2–7 years post-infection [17]. Edlin et. al. and Kunzi et. al. further showed that viruses isolated from individuals who had progressed to AIDS were more IFN resistant than viruses from asymptomatic chronically-infected individuals [45,46]. Likewise, Parrish et. al. proposed that their observation of differences in IFN resistance between TF IMCs and IMCs from unmatched chronically-infected subjects in a subtype B-infected cohort, but not in a subtype C-infected cohort, may have been due to the subtype C-infected donors being sampled at later timepoints in chronic infection [13]. However, it should be noted that we did observe a range of interferon sensitivities across the six transmission pairs, with greater than a 100-fold difference being observed between TF viruses. In future, it would be of interest to determine whether chronically-infected donors in the Zambian discordant couples cohort who failed to transmit infection to their partners harbor more IFN-sensitive viruses than those present in the virus-transmitting donors studied here. However on the basis of the current results it seems likely that IFN-α does not make a major contribution to the HIV-1 transmission bottleneck, or may do so only in some transmission scenarios.

Transmission selection for consensus-like and more neutralization-sensitive TF variants suggests that within-host evolution of HIV-1 in response to human adaptive immune responses may cause a loss of fitness required for the establishment of infection in a naive host following transmission. We show that relatively high *in vitro* replicative capacity and preferential IFN-α resistance were not selected for during transmission of subtype C HIV-1 in the six pairs studied here. Thus, the *in vitro* assays of HIV-1 replication employed here may not be measuring some of the key determinants of transmission fitness, and other models of HIV transmission, such as low dose intravaginal challenges of humanized mice, or human genital explant cultures, may be needed to determine the phenotypic requirements of HIV-1 transmission that genetic differences are pointing to.

## Materials and Methods

### Study subjects

The six HIV-1 subtype C transmission pairs investigated in this study were enrolled in the heterosexual discordant couple cohort at the Zambia-Emory HIV Research Project (ZEHRP) in Lusaka, Zambia. Human subjects protocols were approved by both the University of Zambia Research Ethics Committee and the Emory University Institutional Review Board. HIV-1 serodiscordant couples in this cohort were provided counseling and testing on a monthly basis prior to the negative partner becoming HIV-1 positive. The recipients were enrolled in the International AIDS Vaccine Initiative (IAVI) Protocol C early-infection cohort. Epidemiological linkage was defined by phylogenetic analyses of HIV-1 *gp41* sequences from both partners [47]. All individuals in this study were ART naive during the time of sampling.

### Viral RNA extraction and PCR amplification

Viral RNA extraction and near full-length genome single genome amplification were performed as described in Deymier et al. 2014 [26]. Briefly, viral RNA was extracted from 140μl of plasma using the QIAamp Viral RNA mini kit (Qiagen) and was used for cDNA synthesis carried out with Superscript III (Life Technologies) and an anchored Oligo(dT)_18_ primer. The cDNA was used immediately for PCR amplification. Near full-length single genome PCR amplification was performed by serially diluting cDNA, followed by two rounds of PCR amplification, so that ~30% of wells became positive. Both rounds of PCR were performed in 1x Q5 Reaction Buffer, 1x Q5 High GC Enhancer, 0.35 mM of each dNTP, 0.5 μM of primers and 0.02 U/μl of Q5 Hot Star High-Fidelity DNA Polymerase (NEB) in a total reaction volume of 25 μl. First round primers were, 1U5Cc and 1.3’3’PlCb, and second round primers were 2U5Cd and 2.3’3’plCb [48]. Cycling conditions for both reactions are 98°C for 30s, followed by 30 cycles of 98°C for 10s, 72°C for 7.5min, with a final extension at 72°C for 10min. PCR reactions were run on a 1% agarose lithium acetate gel at 300 V for 25 min in order to determine the presence of a 9 kb band.

### Sequencing

Positive ~9kb single genome amplicons were gel-extracted using the Wizard SV Gel and PCR Clean-Up System (Promega). Purified ~9 kb PCR amplicons were sent for sequencing to the University of Alabama Birmingham (UAB) sequencing core for Sanger sequencing.

In conjunction, multiple amplicons from recipient 3576 were sequenced by single-molecule nucleic acid sequencing (Pacific Biosciences), to confirm the TF [49]. Briefly, SMRTbell libraries were constructed according to the manufacturer's instructions for 10kb amplicons. PCR reactions of DNA amplicons were purified using Wizard SV Gel and PCR Clean-Up System (Promega) and mixed at equal concentrations to a total of 3ug DNA. Library preparation quality was assessed on a Bioanalyzer and SMRT sequencing on the PacBio RSII was performed following primer annealing and P4 polymerase binding to the library preparations. The consensus of the reads, aligned to the HXB2 reference sequence, were then taken to form a TF sequence, which matched the Sanger sequence.

### Sequence analysis

All 9kb viral sequences were aligned in Geneious bioinformatics software (Biomatters, Aukland, NZ) using MUSCLE [50], followed by hand aligning. The Los Alamos National Database HIV Consensus/Ancestral Sequence Alignments were used as reference sequences (http://www.hiv.lanl.gov/content/sequence/NEWALIGN/align.html). Phylogenetic trees were generated using the DIVEIN web server (http://indra.mullins.microbiol.washington.edu/DIVEIN/) [51]. Phylogenetic analyses were performed by maximum likelihood parsimony under Phylogeny/Divergence/Diversity. For nucleotide sequence analysis a general time reversible model was used, with a fixed gamma distribution parameter of 1, and performed with 100 bootstraps. Amino acid phylogenetic analysis was performed using the HIVw model of evolution, with 100 bootstraps [52]. Pairwise distances from each branch node to the subtype C consensus node were extracted from the distance matrices of the phylogenetic trees.

### Generation of full-length IMC

HIV full-length genome infectious molecular clones were generated as described in Deymier et al. 2014 [26]. Briefly, linked recipient specific primers were generated in order to amplify the full long terminal repeat (LTR) from the linked recipient white cell pellet DNA. This LTR was cloned into a pBluescript vector, and the TF sequence of the LTR sequence was inferred as the consensus sequence from multiple clones. Subsequently, a three-piece DNA HD In-Fusion HD cloning (Clontech) ligation reaction using a reamplified clonable near-full length amplicon and two LTR pieces generated by PCR from the linked recipient LTR generated the full-length IMC. TF IMC were correct for the entire genome, whereas NT variants were chimeric for only for the R region of both 5’ and 3’ LTR, which was taken from the TF of that transmission pair. IMC were sequenced in order to confirm a match to the sequence of the single genome amplicon from which it was derived.

### Generation of virus stocks and particle infectivity

293T (American Type Culture Collection) cells were transfected with 1.5μg of plasmid DNA, using the Fugene HD transfection reagent (Roche) according the manufacturer’s protocol. Viral stocks were collected 48 hours post transfection and clarified by centrifugation. These virus stocks were then titered for infectivity on TZM-bl cell, as described previously [53]. The virus stocks were also measured for reverse transcriptase (RT) activity using a radiolabeled reverse transcriptase assay [53]. Particle infectivity of each virus was determined as the ratio of titer (infectious units/μl) over RT signal (RT/μl) for 3 independent experiments. Particle infectivity over time was measured by sampling 8ul (0.4%) per time point over a 3 day period.

### Replication in PBMC and interferon resistance

Frozen peripheral blood mononuclear cells (PBMC) from buffy coats were thawed and stimulated with 20 U/ml of interleukin-2 (IL-2) and 3ug/ml of phytohemagglutinin (PHA) in R10 (Roswell Park Memorial Institute (RPMI) 1640 Medium supplemented with 10% defined fetal bovine serum (FBS), 1 U/ml penicillin, 1ug/ml streptomycin, 300ug/ml L-glutamine) for 72 hours at 37C. After 48 hours, 1,000 IU/ml of interferon-α2a (Sigma Aldrich, Product # SRP4594) was added to a portion of cells 24 hours prior to infection. 1×10^6^ cells were then infected in 15ml conical tubes by 2 hour spinoculation at 2,200 rpm with an MOI of 0.01 based on the TZM-bl titer in triplicate. Cells were then washed twice in 13ml RPMI, resuspended in 500ul of R10 media and plated in a 48 well plate in triplicate. 50ul of supernatant was then sampled every 48 hours starting with a day zero time point taken 2–3 hours after plating to get a baseline reverse transcriptase activity for each infection well using the radiolabeled reverse transcriptase assay.

Where noted in the text, an alternative strategy for another independent experiment with CD8-depleted PBMC was used with a few differences: anti-CD3 (R&D Systems clone UCHT1; 50ng/ml working concentration) and anti-CD28 (eBioscience clone CD28.2; 100ng/ml working concentration) antibodies were used to stimulate MACS microbead (from Miltenyi plus the MACS LD columns) CD8-depleted PBMC from three separate donors in a mixed lymphocyte reaction and then infected at an MOI of 0.1 based on TZM-bl titer. 2×10^5^ cells were then infected in the presence and absence of 5,000 IU/ml of interferon-α2a (Peprotech) and cells were washed three times with 10ml of RPMI and supernatant tested by a modified ELISA assay using the AlphaLISA HIV p24 (high sensitivity) kit (Product # AL291C PerkinElmer) per protocol instructions, using the same media for the standard as in the sample and loading 5ul per well.

The replication score (RC score) for each variant was calculated using a normalized area under the curve. The median of the replicates were background subtracted using the day 2 time point, adjusted for sampling by a measured exponential decay correction, and area under the curves (AUC) were divided by the AUC for a standard lab adapted subtype C virus, MJ4, to compare across transmission pairs analyzed on different days. Interferon-α2a resistance was measured in a similar fashion, followed by calculating the ratio of the RC score in the presence of interferon divided by the RC score in the absence of interferon.

### Monocyte derived dendritic cell infections

Monocyte derived dendritic cells (MDDC) were isolated from two healthy blood donors by CD14 positive bead isolation (Miltenyi Biotec), followed by culture at 37°C in R10, supplemented with 40 ng/ml IL-4 (Peprotech) and 20ng/ml GM-CSF (Peprotech) for 7 days. MDDC differentiation was confirmed by flow cytometry using the following antibodies and stains: α-CD14 PB (clone M5E2), α-CD11c APC (clone S-HCL-3), α-HLA-DR V500 (clone G46-6) (BD Biosciences), and the LIVE/DEAD Fixable Near-IR Dead Cell Stain Kit (Life Technologies). The phenotype of MDDC after 7 days was CD14 low, CD11c high, and HLA-DR high, as expected. Cells were harvested, and 3x10^5^ MDDC were seeded in a flat-bottom 96-well plate. MDDC were infected in a volume of 250μl of R10 with virus at an MOI of 1 for 4 hours. Cells were then washed three times with RPMI, and cultured for 12 days in R10 supplemented with 40 ng/ml IL-4 and 20ng/ml GM-CSF. 50μl of culture supernatant was collected every two days and replaced with fresh media. The supernatant was then analyzed for virus production by the radiolabelled RT assay [53].

### Interferon Elisa

IFN-α levels were measured by the VeriKine Human IFN Alpha ELISA Kit from supernatants 8 days after PBMC and MDDC infections with a subset of viruses from pairs 331 and 3678. The negative controls included media from PBMC and MDDC uninfected cultures. The positive controls included IFN-α spiked media equal to the initial amount of IFN-α utilized in these infections, along with supernatant from an infection carried out in the presence of IFN-α.

### Neutralization assay

IMC derived virus and plasma taken from the same time point in the transmitting partner (donor) along with the TF from the recipient, were used to test antibody neutralization of variants circulating near the time of transmission. The TZM-bl neutralization assay was adapted for use with IMCs, in a similar fashion to what has been published previously for IMC [54] and pseudoviruses [1,28]. Briefly, heat inactivated plasma was serially diluted 5-fold starting at 1:100, and each dilution was then mixed with 20 IU/ul of virus at a 1:1 ratio. After incubation at 37°C for 1 hour, the plasma and virus mixtures were used to infect previously seeded TZM-bl cells (24 hours prior to infection at 6x10^3^ cells per well in a 96-well plate). After a 40 hour incubation, the Promega Reporter Buffer was used to lyse cells according to manufacturer instructions and, following two freeze-thaw cycles, luciferase was measured with the Luciferase Assay System from Promega (Catalog # E1501) in the supernatants on a luminometer using the Gen5 2.00 software. Maximal percent inhibition (compared to the no plasma control) was calculated at a dilution of 1:00 after background subtraction and removal of variants with a signal less than three times background for cell only control wells. The data is averaged from each virus run in duplicate from two independent experiments.

## Supporting Information

S1 Fig Particle infectivity from 293T and PBMC derived virus. (A) Particle infectivity (TZM-bl titer divided by reverse transcriptase activity) of 293T cell derived TF virus stocks at different time points post transfection. (B) Correlation of particle infectivity assessed from day 8 of a PBMC infection and the particle infectivity from 293T derived stocks 48 hours after transfection of a subset of eleven viruses (p < 0.0001, r = 0.9455).
(TIF)

S2 Fig Particle infectivity correlates with replicative capacity. Spearman correlation of particle infectivity and replicative capacity score of all TF & NT virus variants (p = 0.0005, r = 0.5712).
(TIF)

S3 Fig Replication of TF and NT viruses in monocyte derived dendritic cells. Virus growth in monocyte derived dendritic cells was measured by analyzing supernatant reverse transcriptase activity for 12 days following infection. Replication is depicted (y-axis) as the area under the curve for each virus variant. TF (blue) and NT (red) are presented with their group median. The difference between the groups was analyzed using a two-tailed Mann Whitney test (p = 0.87). Results are the average of replication in two healthy donors.
(TIF)

S4 Fig TF and NT resistance to IFN-α. (A) Correlation of IFN-α resistance (RC IFN+/-) and RC Score of variants from Fig 6C (p = 0.0028, r = 0.6467). (B) The TF (blue) and three NT (red, yellow, orange) variants from pair 331 with a representative range of RC scores were tested for replication in the presence of IFN-α concentrations from 0.5 U/ml–10,000 U/ml. Supernatant reverse transcriptase (RT) activity at day 7 post-infection are shown. (C) Analysis of IFN-α levels in day 8 supernatants from PBMC and MDDC infected with a subset of viruses, to test for IFN-α induction *in vitro*. Negative controls are shown in white, MDDC infections in light gray, PBMC infections in dark gray, and positive controls in black. (D) Area under the curve (solid lines) and IFN-α resistance ratios (dotted lines) from infections initiated at a range of MOI for the 3678 TF (blue), along with an NT variant (red) with a different replicative capacity.
(TIF)

S5 Fig Replication of TF and 6-month consensus infectious molecular clones. RC scores of three subtype B TF/6-month virus pairs (described in [17]) in activated PBMC.
(TIF)
