# Supplementary figures and images for: Heterosexual Transmission of Subtype C HIV-1 Selects Consensus-Like Variants without Increased Replicative Capacity or Interferon-α Resistance
Source: PLoS Pathog. 2015 Sep 17;11(9):e1005154. doi: 10.1371/journal.ppat.1005154 (PMC4574710; doi:10.1371/journal.ppat.1005154)

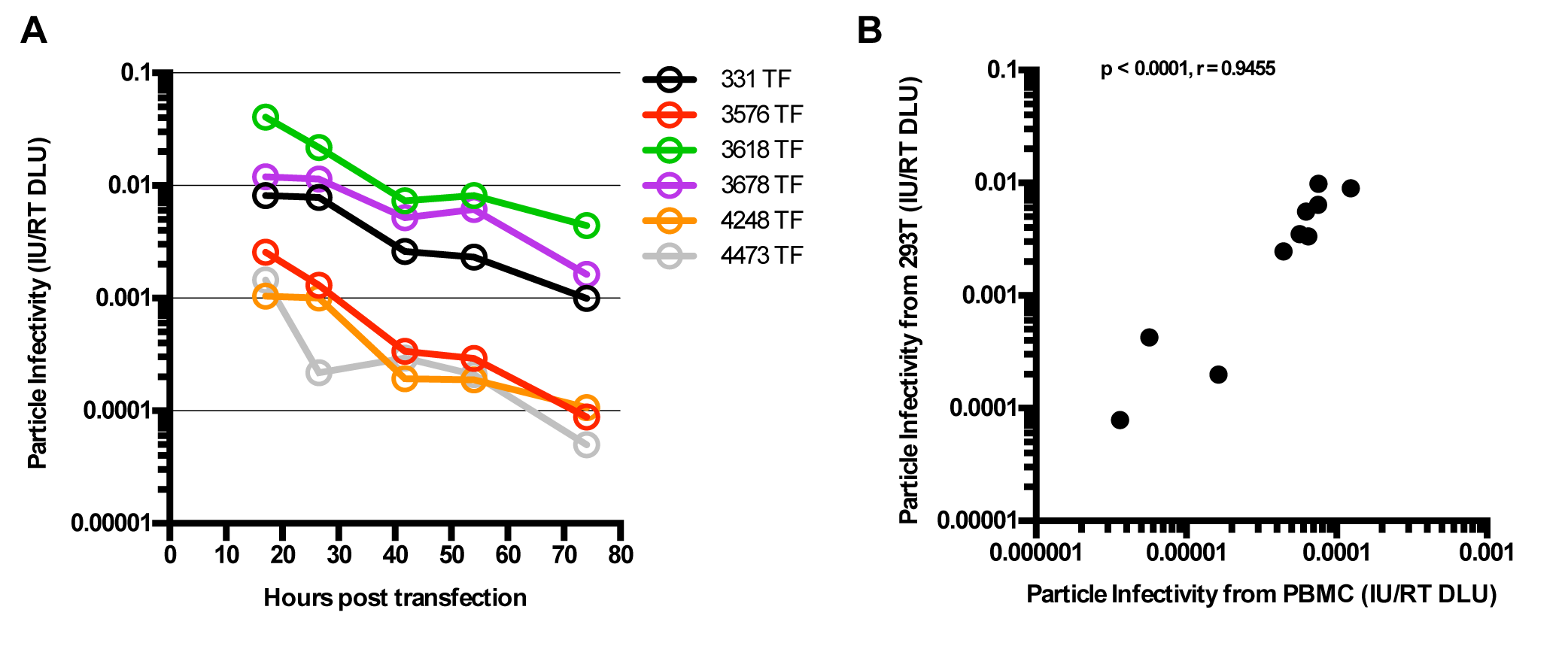

Supplement: S1 Fig — (A) Particle infectivity (TZM-bl titer divided by reverse transcriptase activity) of 293T cell derived TF virus stocks at different time points post transfection. (B) Correlation of particle infectivity assessed from day 8 of a PBMC infection and the particle infectivity from 293T derived stocks 48 hours after transfection of a subset of eleven viruses (p < 0.0001, r = 0.9455). (TIF) [file ppat.1005154.s001.tif]

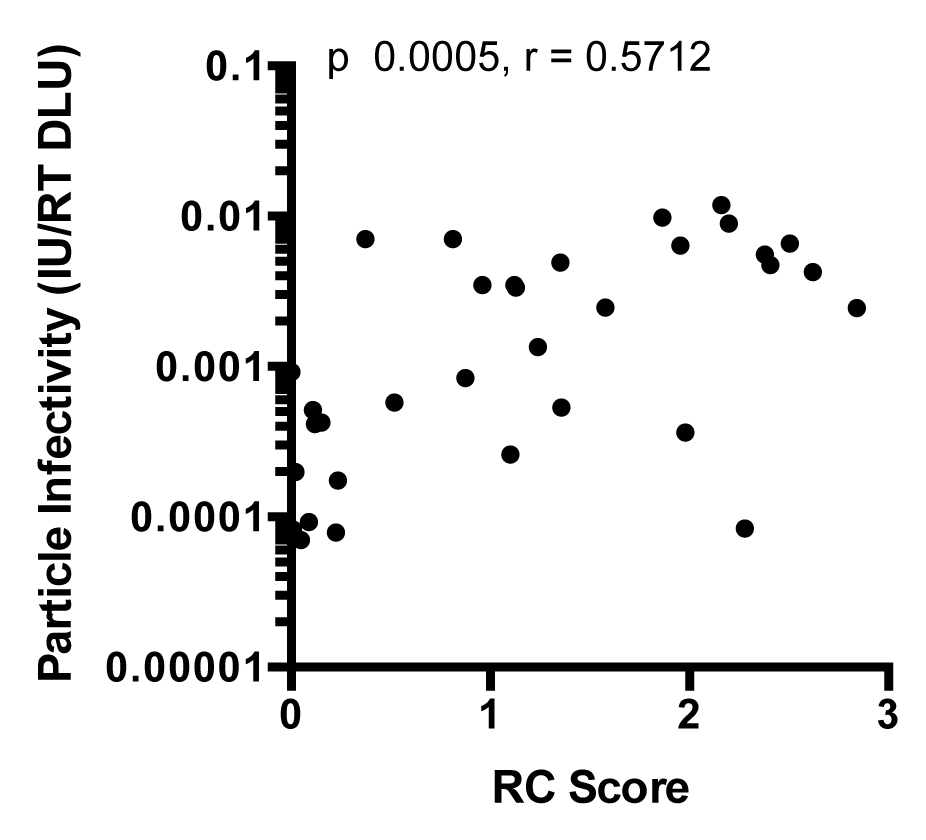

Supplement: S2 Fig — Spearman correlation of particle infectivity and replicative capacity score of all TF & NT virus variants (p = 0.0005, r = 0.5712). (TIF) [file ppat.1005154.s002.tif]

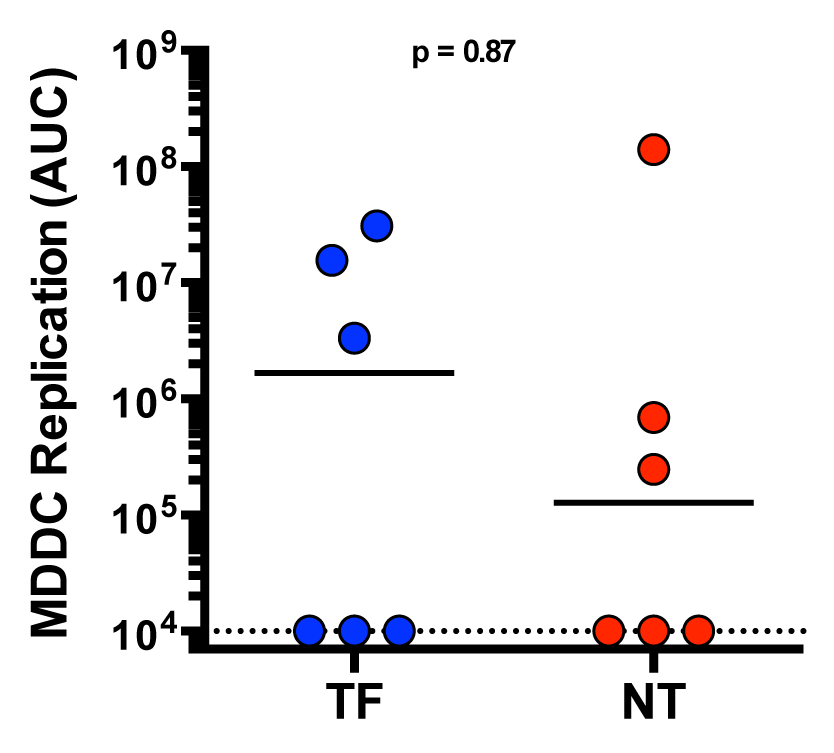

Supplement: S3 Fig — Virus growth in monocyte derived dendritic cells was measured by analyzing supernatant reverse transcriptase activity for 12 days following infection. Replication is depicted (y-axis) as the area under the curve for each virus variant. TF (blue) and NT (red) are presented with their group median. The difference between the groups was analyzed using a two-tailed Mann Whitney test (p = 0.87). Results are the average of replication in two healthy donors. (TIF) [file ppat.1005154.s003.tif]

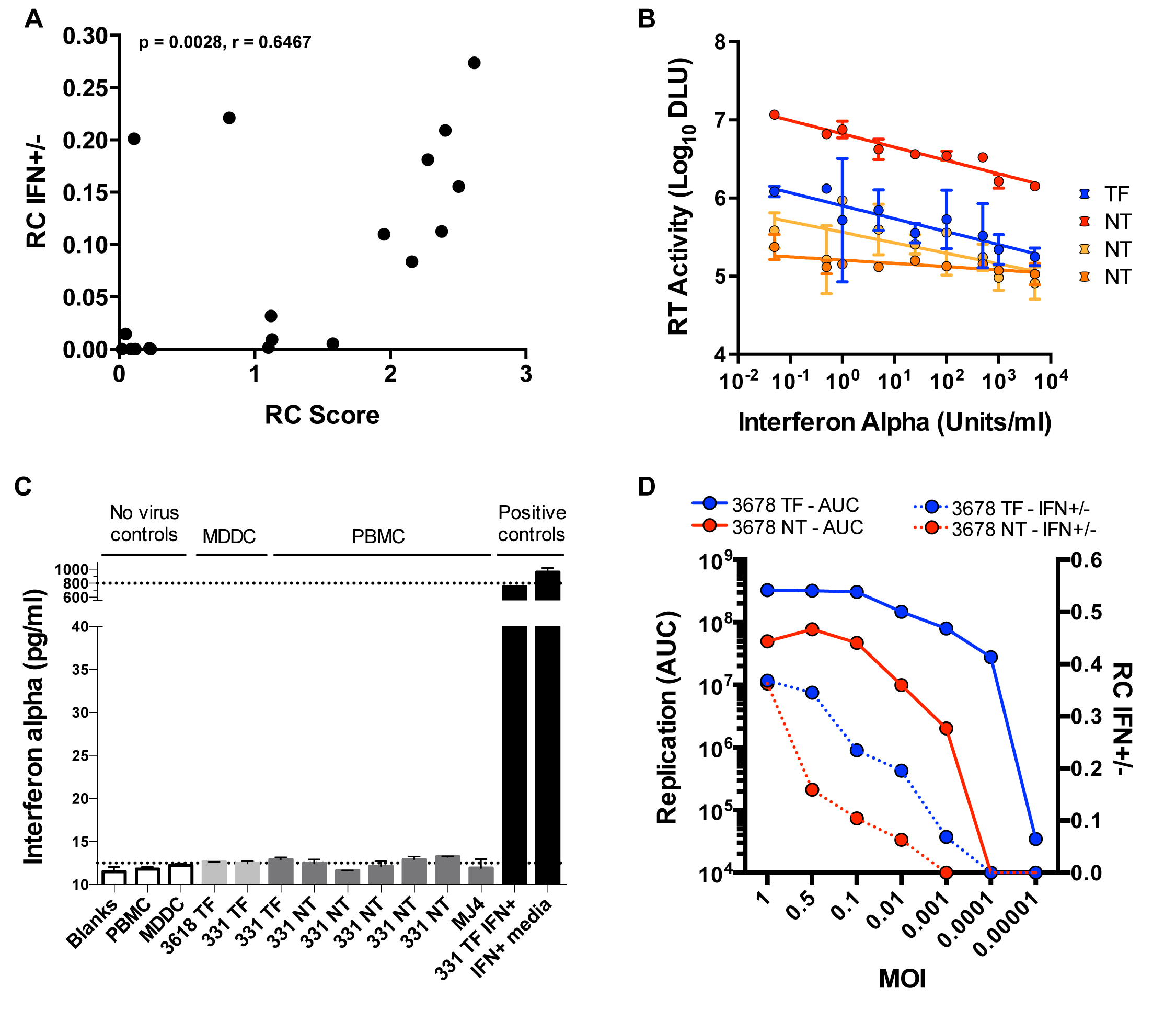

Supplement: S4 Fig — (A) Correlation of IFN-α resistance (RC IFN+/-) and RC Score of variants from Fig 6C (p = 0.0028, r = 0.6467). (B) The TF (blue) and three NT (red, yellow, orange) variants from pair 331 with a representative range of RC scores were tested for replication in the presence of IFN-α concentrations from 0.5 U/ml–10,000 U/ml. Supernatant reverse transcriptase (RT) activity at day 7 post-infection are shown. (C) Analysis of IFN-α levels in day 8 supernatants from PBMC and MDDC infected with a subset of viruses, to test for IFN-α induction in vitro. Negative controls are shown in white, MDDC infections in light gray, PBMC infections in dark gray, and positive controls in black. (D) Area under the curve (solid lines) and IFN-α resistance ratios (dotted lines) from infections initiated at a range of MOI for the 3678 TF (blue), along with an NT variant (red) with a different replicative capacity. (TIF) [file ppat.1005154.s004.tif]

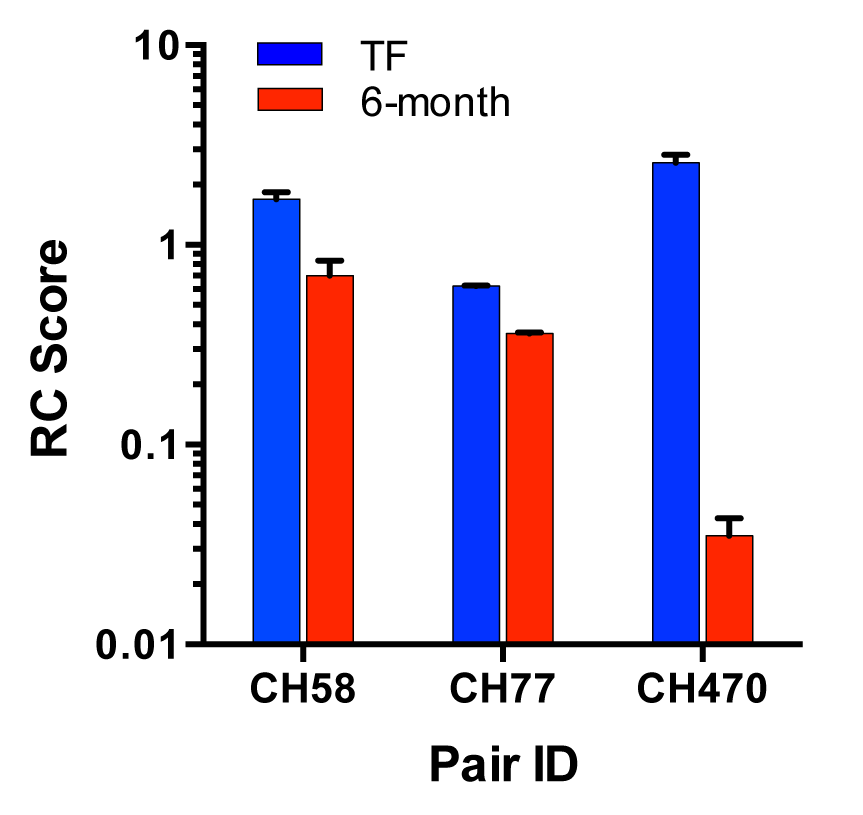

Supplement: S5 Fig — RC scores of three subtype B TF/6-month virus pairs (described in [17]) in activated PBMC. (TIF) [file ppat.1005154.s005.tif]
